# Restriction of HIV-1 Genotypes in Breast Milk Does Not Account for the Population Transmission Genetic Bottleneck That Occurs following Transmission

**DOI:** 10.1371/journal.pone.0010213

**Published:** 2010-04-20

**Authors:** Laura Heath, Susan Conway, Laura Jones, Katherine Semrau, Kyle Nakamura, Jan Walter, W. Don Decker, Jason Hong, Thomas Chen, Marintha Heil, Moses Sinkala, Chipepo Kankasa, Donald M. Thea, Louise Kuhn, James I. Mullins, Grace M. Aldrovandi

**Affiliations:** 1 Department of Microbiology, University of Washington, Seattle, Washington, United States of America; 2 Childrens Hospital of Los Angeles, Los Angeles, California, United States of America; 3 Boston University School of Public Health, Boston, Massachusetts, United States of America; 4 University of Southern California Keck School of Medicine, Los Angeles, California, United States of America; 5 Department of Microbiology, University of Alabama at Birmingham, Birmingham, Alabama, United States of America; 6 Lusaka District Health Management Team, Lusaka, Zambia; 7 University Teaching Hospital, University of Zambia, Lusaka, Zambia; 8 Columbia University, New York, New York, United States of America; 9 Department of Medicine, University of Washington, Seattle, Washington, United States of America; University of California San Francisco, United States of America

## Abstract

**Background:**

Breast milk transmission of HIV-1 remains a major route of pediatric infection. Defining the characteristics of viral variants to which breastfeeding infants are exposed is important for understanding the genetic bottleneck that occurs in the majority of mother-to-child transmissions. The blood-milk epithelial barrier markedly restricts the quantity of HIV-1 in breast milk, even in the absence of antiretroviral drugs. The basis of this restriction and the genetic relationship between breast milk and blood variants are not well established.

**Methodology/Principal Findings:**

We compared 356 HIV-1 subtype C gp160 envelope (*env*) gene sequences from the plasma and breast milk of 13 breastfeeding women. A trend towards lower viral population diversity and divergence in breast milk was observed, potentially indicative of clonal expansion within the breast. No differences in potential N-linked glycosylation site numbers or in gp160 variable loop amino acid lengths were identified. Genetic compartmentalization was evident in only one out of six subjects in whom contemporaneously obtained samples were studied. However, in samples that were collected 10 or more days apart, six of seven subjects were classified as having compartmentalized viral populations, highlighting the necessity of contemporaneous sampling for genetic compartmentalization studies. We found evidence of CXCR4 co-receptor using viruses in breast milk and blood in nine out of the thirteen subjects, but no evidence of preferential localization of these variants in either tissue.

**Conclusions/Significance:**

Despite marked restriction of HIV-1 quantities in milk, our data indicate intermixing of virus between blood and breast milk. Thus, we found no evidence that a restriction in viral genotype diversity in breast milk accounts for the genetic bottleneck observed following transmission. In addition, our results highlight the rapidity of HIV-1 *env* evolution and the importance of sample timing in analyses of gene flow.

## Introduction

Studies of HIV-1 variants in blood indicate that regardless of transmission route, descendents of a single virion establish infection in the new host [1], [2], [3], [4], [5], [6], [7], [8]. Transmitted viruses are also distinguished by almost exclusive use of the CCR5 co-receptor, and non-subtype B founder strains have envelopes with shorter variable loops and fewer N-linked glycosylation sites [6], [8], [9], [10], [11], [12], [13]. The factors that govern this selection are unknown. Most transmissions occur across mucosal surfaces lined by highly selective epithelial barriers, which produce a variety of factors contributing to a distinct immunologic milieu. Levels of HIV-1 in these transmitting compartments (e.g., genital fluids and breast milk) are usually much lower than those in blood [14], [15], [16], but few studies have addressed how mucosal restriction contributes to the apparent transmission genetic bottleneck. Elucidating the relationship between HIV-1 strains circulating in blood and those in mucosal transmitting compartments is important for understanding the dynamics of transmission as well as for the design of vaccines and other prevention strategies [17].

Breast milk transmission remains a major source of pediatric HIV-1 infection particularly in sub-Saharan Africa, where 90% of pediatric HIV-1 infections occur [18], [19]. The content of milk is dynamically and tightly regulated; the types and activation state of breast milk cells, as well as antibodies, cytokines, and chemokines, are distinct from contemporaneously obtained blood [20], [21]. Even in the absence of antiretroviral therapy, the amount of HIV-1 in breast milk is usually 10–100 fold less than that present in plasma, which suggests limited exchange of virus between these two sites [14]. The degree of immunologic and biochemical compartmentalization between blood and milk strongly suggests that HIV-1 strains would also be compartmentalized, i.e., there would be a restriction of viral passage (and consequent gene flow) [22]. Tissue compartmentalization has been reported for other sites such as cerebral spinal fluid, brain, the male and female genital tracts, lymphoid cells, blood monocytes, and the lung [23], [24], [25], [26], [27], [28], [29]. Evidence for virologic compartmentalization in the colostrum has been found in small ruminants [30] and SIV [31], but human studies involving viral compartmentalization in breast milk have been limited and contradictory [32], [33], [34], [35].

We sought to characterize the extent to which breast milk variants were distinct from viruses circulating in the blood. We compared viral diversity and divergence between blood and breast milk, as well as viral populations between the right and left breast. We assessed whether breast milk was enriched for a “transmissible” viral phenotype, i.e., CCR5-tropic variants, and as previously suggested for non-subtype-B transmissions, with shorter variable loops and fewer potential N-linked glycosylation sites [6], [8], [9], [10], [11], [12], [13]. We also compared the susceptibility of plasma and breast milk HIV envelopes to two entry inhibitors. Finally, given HIV's extraordinary evolutionary rates and the inherent difficulties in obtaining samples at the time of transmission, we investigated the relationship between sampling time and virologic compartmentalization using five distinct algorithms.

## Materials and Methods

### Subject enrollment, sample collection, and processing

Samples were obtained from 13 subjects participating in the Zambia Exclusive Breastfeeding Study (ZEBS) (Table 1). ZEBS was a randomized clinical trial designed to assess the impact of short-term exclusive breastfeeding on HIV-1 transmission and child mortality [19], [36]. All women signed informed consent. ZEBS was approved by Human Subjects Committees at the investigators' institutions in the US (Boston University, Columbia University, University of Alabama, Birmingham and Childrens Hospital Los Angeles) and by the University of Zambia Research Ethics Committee. Laboratory specimens were completely anonymized and unlinked.

**Table 1:** CD4+ T cell count at time of study entry, viral load at sequence sample time, and number of unique gp160 sequences.

Subject ID	# Days between PL and BM sampling^a^	CD4+T cells	Plasma Viral Load (copies/mL)	BML^b^ Viral Load (copies/mL)	BMR^c^ Viral Load (copies/mL)	# PL sequences	# BML sequences	# BMR sequences
31	0	163	131,175	4,111	2,971	11	2	8
21	0	55	242,813	20,687	14,726	10	8	13
32	0	134	235,196	3,987	10,570	13	16	10
7	0	317	75,933	25,694	17,625	12	12	8
33	0	82	437,545	1,294	1,885	13	7	10
10	0	300	663,850	23,862	387	9	11	1
34	10	172	150,449	32,131	11,274	9	8	10
17	15	291	51,349	3,448	7,518	15	5	0
35	31	419	100,187	11,940	24,440	13	6	4
14	43	94	375,319	637	1,538	13	8	9
1	102	76	300,000	11,340	74,970	9	5	6
3	115	94	206,763	3,272	3,019	11	2	10
16	141	118	211,792	20,207	18,277	17	10	12

a PL always sampled previous to BM.

b BML  =  Milk from the Left Breast.

c BMR  =  Milk from the Right Breast.

All of the women received single-dose nevirapine (sdNVP) peripartum, but were otherwise antiretroviral drug (ARV)-naïve. Plasma and peripheral blood mononuclear cells (PBMC) were separated from whole blood by centrifugation. Milk collected separately from both breasts was centrifuged and the cell-free supernatant analyzed [37]. None of the women had signs or symptoms of mastitis prior to or at the time of breast milk collection.

HIV-1 RNA levels in plasma (PL) were determined by the Roche Amplicor assay, while breast milk levels were determined by the Roche Ultrasensitive assay (Roche Diagnostics, Branchburg, New Jersey), which we previously validated for HIV quantification in breast milk (BM) [37]. Breast milk sodium concentrations were measured with an ion-selective electrode (Beckman Coulter Synchron LX20: Beckman Coulter, Fullerton, CA).

### RNA extraction, reverse transcription, PCR, cloning, and sequencing

Amplification, cloning, and sequencing of complete gp160 *env* from PL and milk from each breast were performed as previously described [6] with the following modifications. In order to avoid resampling of the same viral template after PCR, multiple independent PCRs were performed at limiting dilution or near-limiting dilution conditions and only one clone from each PCR was used in the analysis [38], [39]. All sequences were checked for cross-contamination via ViroBLAST [40] against published and local databases, and by observing that sequences from each subject clustered separately from every other subject in a Jukes-Cantor phylogenetic tree calculated in Seaview [41] using an alignment of all sequences from all subjects. No evidence of sample mix-up or contamination was observed (data not shown).

### Phylogenetic analysis

Nucleotide sequences were aligned with MUSCLE v3.7 [42] and refined manually within MacClade v4.08 software (Sinauer Associates, Inc., Sunderland, MA). Four subtype C reference sequences (accession numbers AY772699, U52953, U46016, and AF067155) were included in each subject's alignment for use as an out-group to root the trees. Ambiguously aligned regions due to extreme variability were excluded when calculating phylogenetic trees. Maximum likelihood trees were calculated in PhyML [43] using the online tool DIVER (http://indra.mullins.microbiol.washington.edu/cgi-bin/DIVER/diver.cgi), which implemented the evolutionary model GTR+I+G for all subjects. Diversity of viral sequences for each tissue within each subject was calculated in DIVER as pairwise distances under the previously estimated maximum likelihood model between all sequences within each tissue. Divergence of viral sequences for each tissue within each subject was calculated as the genetic distance between each sequence and the most recent common ancestor (MRCA) of the examined sequences, as calculated in DIVER. Statistical comparisons between PL and BM diversity were performed using the two-sample tests for comparing intra-individual sequence diversity between populations [44] (http://www.scharp.org/users/adecamp/diverstest/runtests.php); comparisons within each individual were calculated using the T_poolmedian_ test, which accounts for the multiple comparisons inherent in a pairwise diversity matrix, while the comparison between PL and BM among all of the subjects pooled was performed with the T_subjmean_ test, which treats the averages of the pairwise distances within each individual (accounting for multiple comparisons) as the observations. Divergence comparisons were made using the Wilcoxon Rank Sums test for within-individual comparisons. A generalized estimating equations (GEE) model with exchangeable correlation matrix was used for the pooled BM vs. PL divergence comparison, which accounted for repeated measures from multiple individuals. Shannon entropy scores [45] were calculated for each position in the protein alignment using the Entropy2 software (http://www.hiv.lanl.gov/content/sequence/ENTROPY/entropy.html).

### Tests for compartmentalization

Five methods were used to determine viral sequence compartmentalization between PL and BM variants [46], [47], [48], [49]. Four of the tests were based on the topology of the phylogenetic trees; one test relied on genetic distances between sequences. The four phylogenetically-derived methods for detecting compartmentalization were: (1) Slatkin-Maddison (SM), which determines the minimum number of migration events between two populations based on the tree topology; (2) Simmonds Association Index (AI), which assesses the degree of population structure, weighting the contribution of each internal node based on how deep it is in the tree, and; Correlation Coefficients, either by (3) length of branches “r” or (4) by number of branches “r_b_”. The correlation coefficients tests examine any two sequences in a tree to determine whether or not they originate from the same compartment by examining tree structure and distances, i.e., the cumulative genetic distances between sequences (the length of branches) (r), or the number of tree branches separating the sequences (r_b_). The distance-based method used was the Nearest Neighbor statistic (Snn), a measure of how often the “nearest neighbor,” or sequence with the shortest distance, from any given sequence is from the same tissue. Permutation tests of 1000 randomizations were performed for each type of analyses and p-values were calculated. Statistics and compartmentalization tests were implemented in HyPhy as described [50], [51].

We also screened each alignment for recombination, since this could confound compartmentalization [50]. For each subject in which no compartmentalization was observed, we used a genetic algorithm approach [52] implemented as the GARD tool in DataMonkey (http://www.datamonkey.org/) to detect recombination breakpoints. Each non-recombinant fragment defined by these breakpoints was then analyzed separately for compartmentalization using the previous methods.

We plotted the individual Snn score, Association Index, correlation coefficients r and r_b_, as calculated above for each subject, versus the number of days between PL and BM sampling, and determined whether there was a linear correlation between these values and the interval using the Spearman's Rho test. Aligned gp160 protein sequences from PL and BM were also analyzed for tissue-specific amino acids using the Viral Epidemiology Signature Pattern Analysis (VESPA) [53] (http://www.hiv.lanl.gov/content/sequence/VESPA/vespa.html).

### Envelope V3 loop genotypic prediction of NSI/SI phenotype

The V3 loop region was analyzed to predict syncytium-inducing phenotype via the subtype-C-specific Web PSSM [54] (http://indra.mullins.microbiol.washington.edu/webpssm/).

### Potential N-Linked glycosylation sites (PNGS) and amino acid lengths

N-linked glycosylation sites were predicted using N-glycosite [55] (http://www.hiv.lanl.gov/content/hiv-db/GLYCOSITE/glycosite.html). The number of amino acids in full-length gp160 and within specific regions of gp120 was tallied for each sequence. Statistical comparisons between PL and BM in each individual were calculated using the Wilcoxon Rank Sums test, while statistical comparisons between PL and BM for pooled data were performed using a Generalized Estimating Equations (GEE) model accounting for repeated measures from multiple subjects.

### Nucleotide sequence accession numbers

All sequences were submitted to GenBank under accession numbers HM036739-HM37037, GU939062-GU939098, GU939143-GU939146, GU939148, GU939150-GU939154, and GU939162-GU939171.

### Phenotypic analysis of BM and PL Env

PL and BM full length Env from 11 women (5 of whom had PL and BM sequences which initially scored as compartmentalized under the previously mentioned tests) were compared for their sensitivity to the entry inhibitors Tak-779 and T-20 using the TZM-bl single-cycle pseudotype assay as previously described [56].

## Results

### Levels of HIV-1 in BM compared to PL

**Figure 1:**
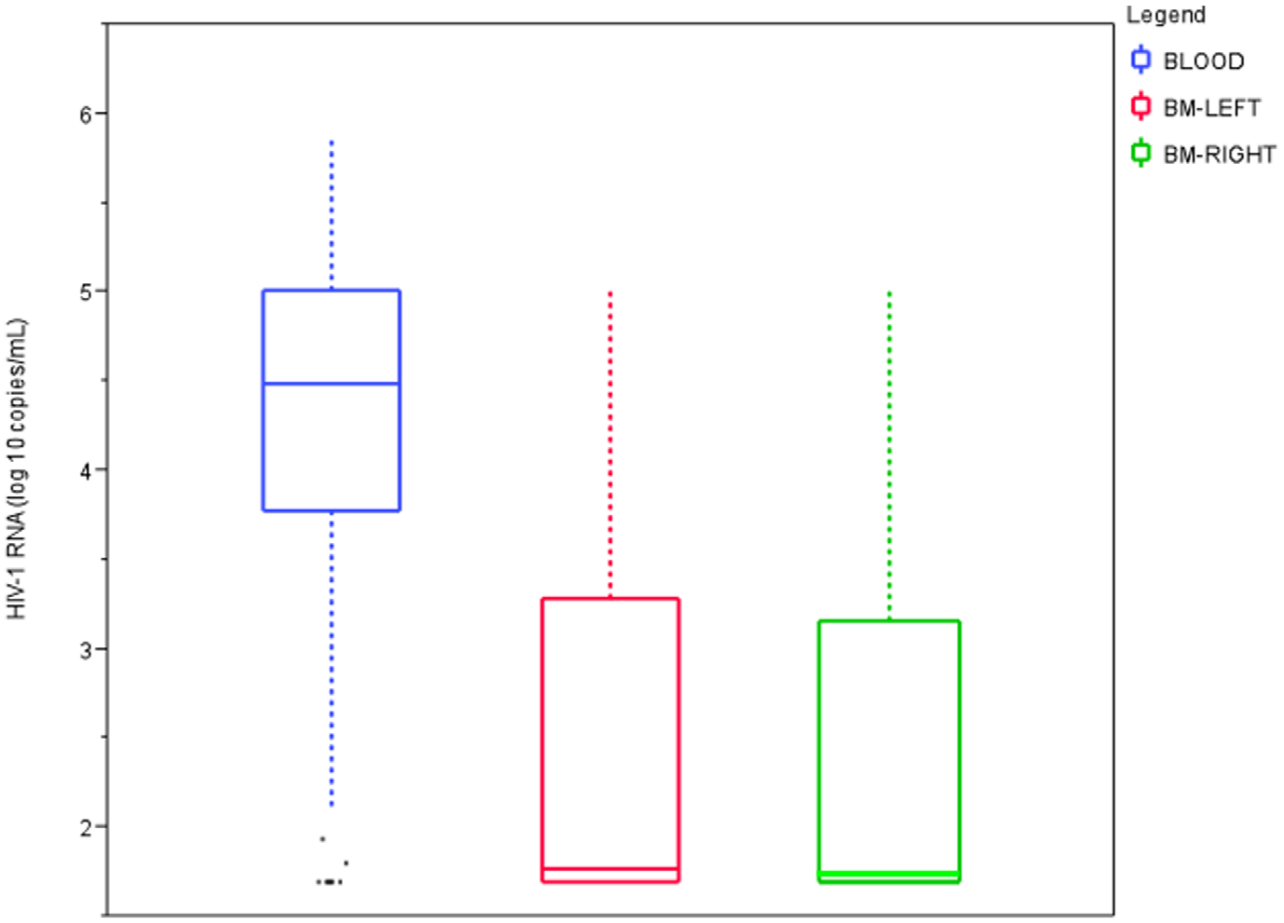
Plasma and breast milk viral load. Viral loads determined by Roche Amplicor (PL) and Roche Amplicor Ultrasensitive (BM) assays. Gray lines are means.

To define the degree to which the breast epithelium restricted the amount of HIV-1 in milk, we compared the amount of viral RNA in PL and BM in over 600 lactating women (Figure 1). As shown in Figure 1, BM HIV-1 RNA was on average 1.8 logs lower than that in PL.

### Subjects and samples

To compare the genetic characteristics of BM and PL HIV-1 *env*, we amplified and cloned full-length gp160 genes from both tissues in chronically HIV-1 subtype C infected women [19], [36]. The clinical characteristics of these women are summarized in Table 1. A total of 356 full-length gp160 sequences from the 13 women were obtained. Although we attempted to amplify at least 10 clones per tissue site, the low amplifiable copy number of viral RNA from BM precluded reaching this goal in some instances. This, along with specimen availability, also resulted in non-contemporaneous sampling in some instances. Sequences from contemporaneous PL and BM samples were collected from six of the subjects (Subjects 31, 21, 32, 7, 33, and 10), while there was an interval of between 10 and 141 days between PL and BM collection in the other 7 subjects (Subjects 34, 17, 35, 14, 1, 3, and 16) (Table 1). Milk samples from right and left breast were collected at the same time in all subjects. All subjects were exclusively breastfeeding at the time of sample collection.

### Phylogenetic analysis of HIV-1 compartmentalization in BM

Viral variants in PL were compared to those in BM from the right and left breast. In four individuals, two or fewer sequences were obtained from either breast, preventing further comparison. In the remaining nine women, BM viral populations were phylogenetically indistinguishable between the left and right breast, regardless of whether there was compartmentalization between PL and BM (data not shown). Analysis of BM variants (11 BML and 10 BMR) obtained at a separate time point in Subject 17 also revealed no difference (sequences from this time point were not used in any other analysis). Thus, we grouped sequences from the right and left breasts together and used all available BM sequences from each individual for the remaining analyses.

**Figure 2:**
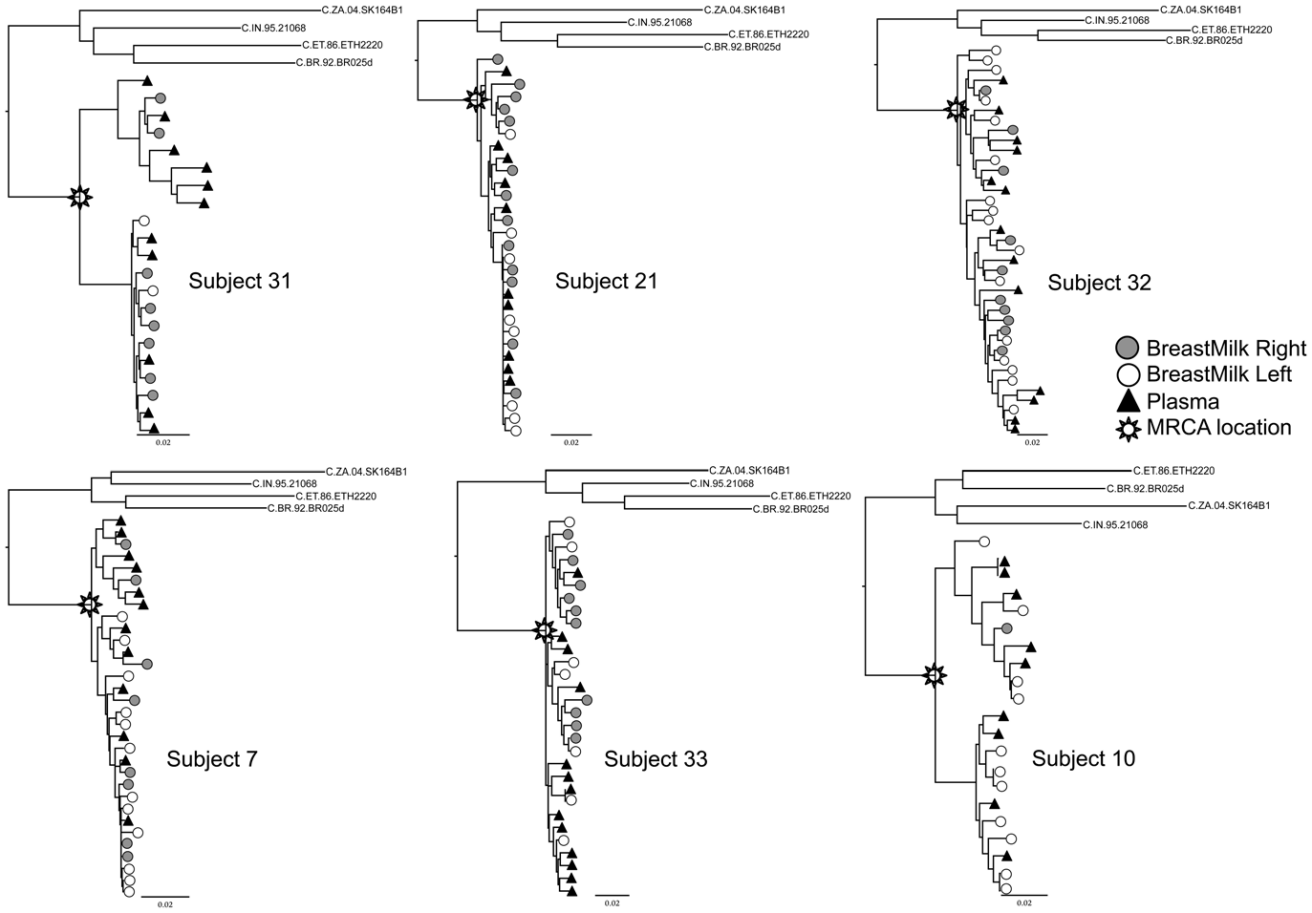
Maximum likelihood trees of region gp160, PL and BM samples obtained contemporaneously. All trees were calculated under the GTR+G+I model, rooted with 4 subtype C reference sequences obtained from LANL sequence database. In all subjects, HIV-1 RNA sequences from the left breast (white circles) and from the right breast (gray circles) were intermixed. The scale at the bottom left of each tree corresponds to the number of substitutions per site (for example, 0.01 = 1 substitution per 100 sites).

**Figure 3:**
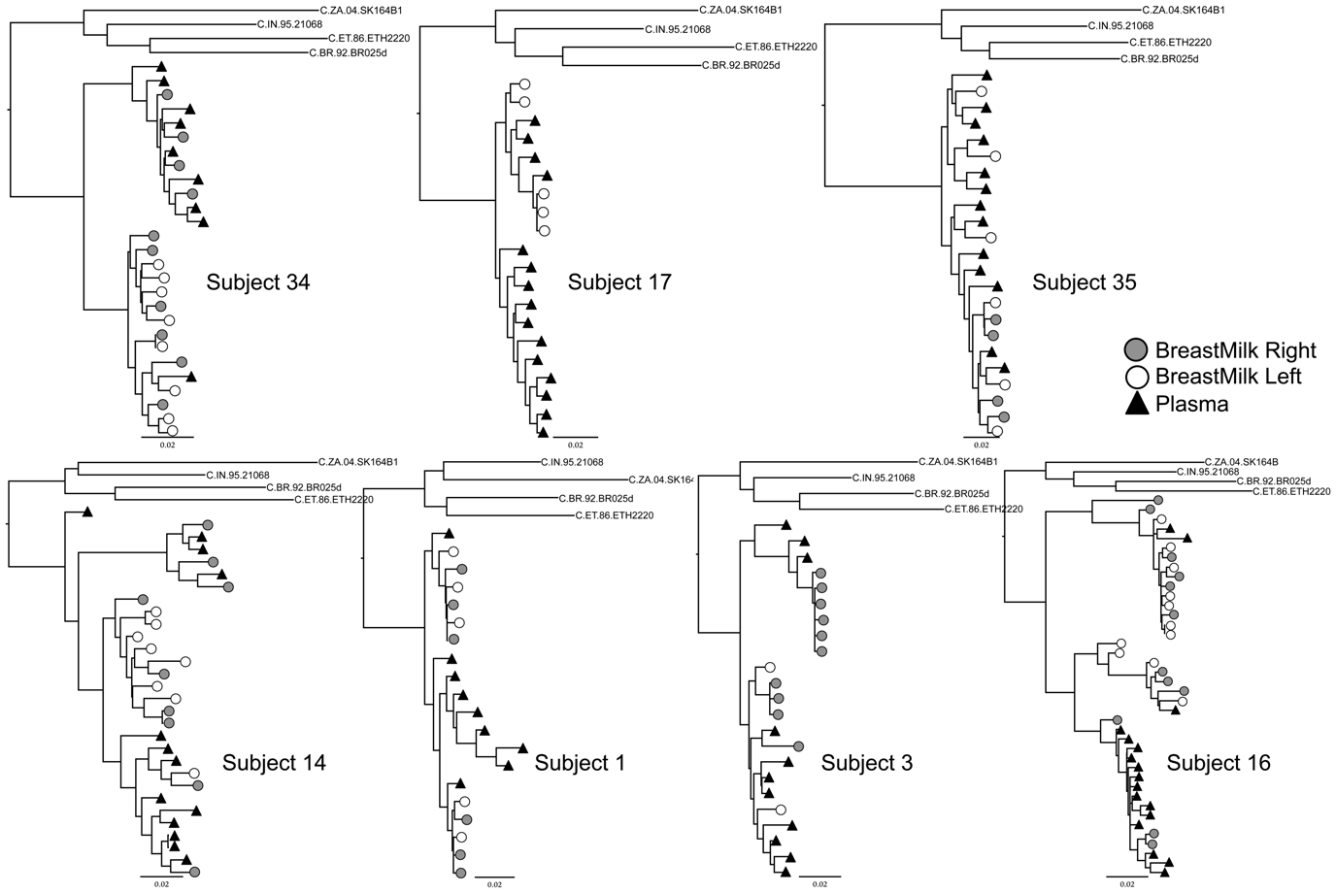
Maximum likelihood trees of region gp160, PL samples obtained previous to BM samples. All trees were calculated under the GTR+G+I model, rooted with 4 subtype C reference sequences obtained from LANL sequence database. In all subjects, HIV-1 RNA sequences from the left breast (white circles) and from the right breast (gray circles) were intermixed. The scale at the bottom left of each tree corresponds to the number of substitutions per site (for example, 0.01 = 1 substitution per 100 sites).

Maximum likelihood trees were calculated (Figures 2 and 3) and datasets were analyzed for compartmentalization. For each subject, if the compartmentalization classifications determined by the different methods were not concordant, we took the majority consensus approach as previously described [51]. Sequences from only one out of the six contemporaneously sampled subjects (Subject 10) were classified as compartmentalized under these criteria (Table 2), and examination of the trees by eye shows sequences from tissues to be heavily intermixed in most of these subjects (Figure 2). However, six out of the seven non-contemporaneously-sampled subjects were classified as compartmentalized (Table 2), consistent with the patterns observed in the trees (Figure 3), suggesting significant viral evolution over relatively short intervals. These data strongly indicate that compartmentalization analyses be performed on contemporaneous samples. When we analyzed non-recombinant fragments (as defined by GARD) separately, results were the same, except in non-contemporaneously-sampled Subject 35 sequences, in which compartmentalization was detected in two of six breakpoint-delineated fragments (data not shown).

**Table 2:** Results of different compartmentalization tests.

Subject ID	Sampling interval (days)	aSM	br	cr_b_	dAI	eSnn
31	0	0.232	0.113	0.065	0.758	0.670
21	0	0.118	0.885	0.469	0.969	0.022
32	0	0.102	0.001	0.029	0.393	0.118
7	0	0.372	0.003	0.016	0.943	0.142
33	0	0.008	0.008	0.007	0.449	0.707
10	0	0.648	0.409	0.774	0.466	0.259
34	10	0.024	0.001	0.003	0.386	0.018
17	15	0.003	0.015	0.004	0.038	<0.001
35	31	0.162	0.158	0.036	0.839	0.185
14	43	0.006	0.040	0.002	0.292	<0.001
1	102	<0.001	0.001	0.001	0.005	<0.001
3	115	<0.001	0.066	0.038	0.008	0.005
16	141	<0.001	0.001	0.001	0.001	<0.001

Values are p-values obtained from randomization tests. P<0.05 was considered evidence of compartmentalization.

a SM  =  Slatkin-Maddison.

b r  =  Correlation coefficient by length of branches.

c r_b_  =  Correlation coefficient by number of branches.

d AI  =  Association Index.

e Snn  =  Nearest neighbor statistic.

**Figure 4:**
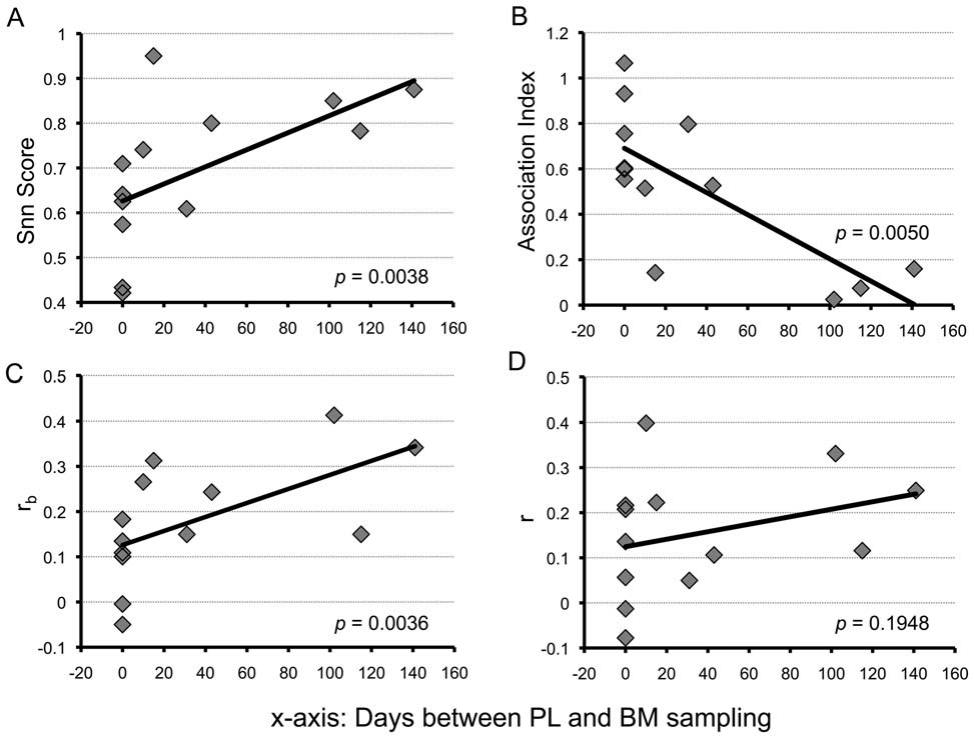
Correlation between sampling interval and measures of compartmentalization. (**A**) Snn score vs. sampling interval; Snn scores close to 1 indicate segregated populations, while scores close to 0.5 indicate mixed populations. (**B**) Simmonds Associative Index (AI) vs. sampling interval; the AI is based on a grouping score (weighted by position in the tree) in which higher AI  =  less grouping of sequences from same tissue in the tree. (**C and D**) Generalized correlation coefficient r_b_ and r vs. sampling interval; r_b_ and r offer a way to correlate the number of internal nodes (r_b_) or branch length (r) between two sequences in a tree with the information about whether or not they were isolated from the same compartment. P-values from Spearman's Rho tests indicate significant linear correlations in A, B, and C.

To further highlight the importance of contemporaneous sampling in compartmentalization testing, we found a correlation between the number of days between PL and BM sampling and several qualitative measures of compartmentalization (Figure 4). We plotted these values against the number of days between PL and BM sampling for all 13 subjects and found a significant linear correlation between the sampling interval and the Snn score, the AI, and the correlation coefficient r_b_. This further demonstrates that non-contemporaneously sampled subjects should not be evaluated for compartmentalization, as the results are likely to be confounded by viral evolution during the sampling interval.

### Sequence diversity and divergence of PL and BM HIV-1 populations

**Figure 5:**
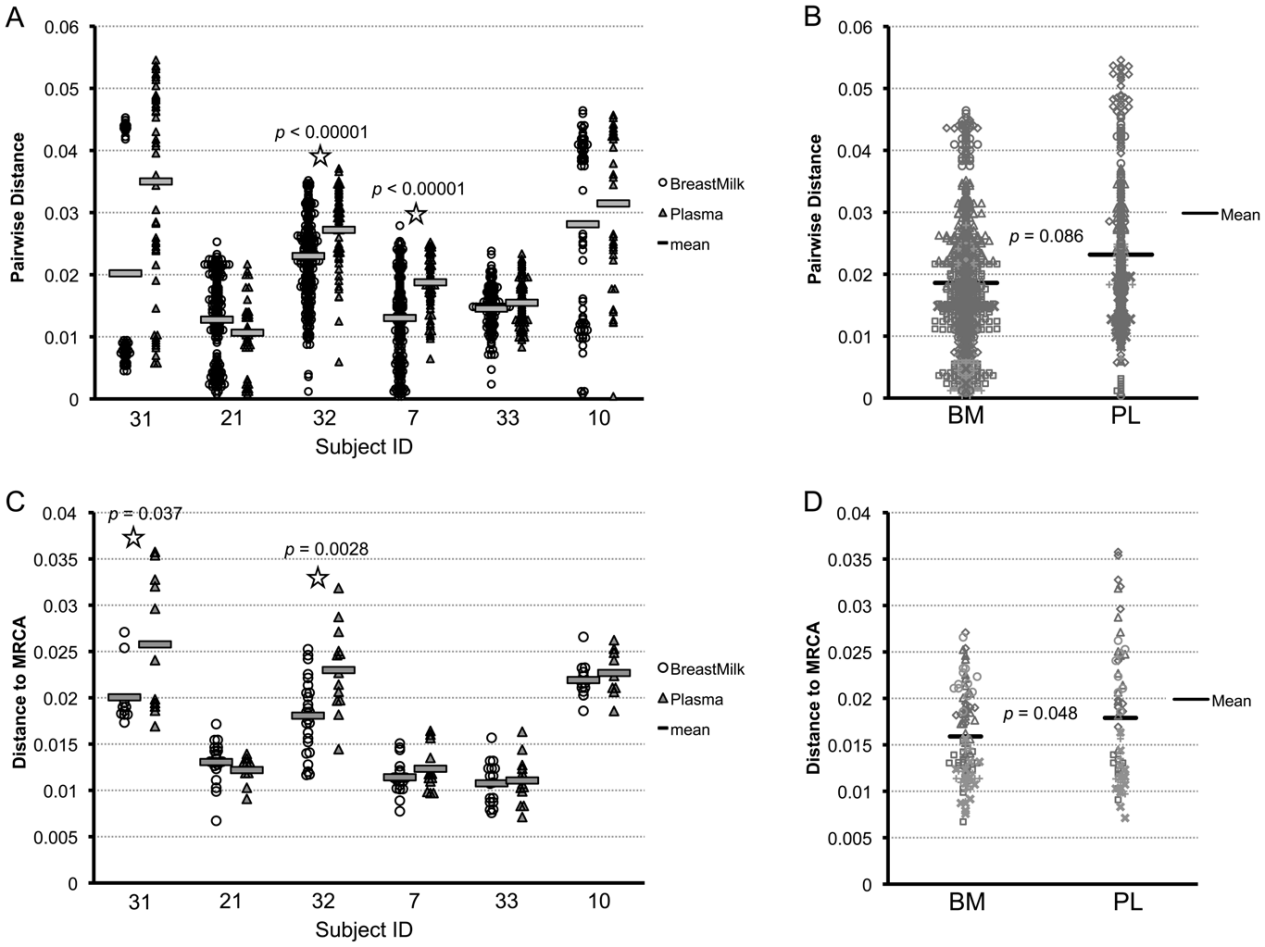
Diversity and divergence in breast milk and plasma. (**A**) Comparing mean diversity of virus in breast milk and plasma HIV-1 RNA gp160 sequences of contemporaneously sampled subjects. Triangles  =  plasma, circles  =  breast milk; star  =  significant difference between plasma and breast milk diversity, p-values from Wilcoxon rank sums test. (**B**) Comparing diversity in breast milk and plasma in all contemporaneously sampled patients in aggregate, using Gilbert, Rossini, and Shankarappa's method for comparing intra-individual genetic sequence diversity between populations. Black lines are means. (**C**) Comparison of mean divergence of virus in BM and PL within contemporaneously sampled subjects. P-values from Wilcoxon rank sums test. (**D**) Comparison of viral divergence between the BM and PL of all contemporaneously sampled subjects in aggregate. P-value obtained using a GEE model. Black lines are means.

We examined pairwise genetic diversity and divergence from the subjects' MRCA in the six contemporaneously sampled subjects only. The node of the tree at which the MRCA was calculated for each subject is indicated in Figure 2. These analyses were not performed on non-contemporaneously obtained samples since data on length of infection and other confounders were not available. Nucleotide diversity between tissues was significantly different in two out of the six subjects (Subjects 32 and 7), and in both of these individuals, PL exhibited higher diversity compared with BM (Figure 5A). We pooled all subjects' PL diversity values and compared them to all subjects' BM diversity values and found no significant difference overall, though there was a trend for BM having less diversity than PL (p = 0.086) (Figure 5B).

We calculated the genetic distance from each sequence to the MRCA as a measure of potential viral evolution and compared BM to PL. In two subjects, PL was significantly more divergent from the MRCA than BM (Subjects 31 and 32) (Figure 5C). When pooling all subjects' PL divergence values and comparing them to all subjects' BM divergence values, BM was less divergent than PL overall (p = 0.048) (Figure 5D).

The extent of amino acid (AA) variability was measured using site-specific Shannon Entropy scores [53]. Subject-specific patterns differed between PL and BM, most often in regions of extreme variability and ambiguous alignment; however, no consistent pattern in AA variability across individuals was identified. Likewise, when we looked for signature motifs by calculating the frequency of AA at each site using VESPA, we identified intra-individual signature sites distinguishing PL and BM but no inter-host signature pattern was found.

### Potential N-Linked Glycosylation in PL and BM HIV-1 populations

We counted the number of potential N-linked glycosylation (PNG) sites in PL and BM clones. No significant differences were observed in the total number of PNG over the entire gp160 region, except in one subject where fewer PNG were observed in milk compared to plasma (Subject 34) (data not shown). When the analysis was restricted to the V1 to V4 region, where most PNG occur, four subjects had significantly fewer PNG in BM than in PL (Subjects 34, 17, 1, and 3), while one subject had significantly more PNG in BM than in PL (Subject 16) (data not shown). However, when examined in aggregate, no difference in the number of BM and PL PNG were observed in gp160. The same held true when each region (V1, V2, C2, V3, C3, V4, C4, V5, and V1 to V4) was analyzed separately.

### Length of variable regions of HIV-1 *env* in PL and BM

We counted the number of amino acids in gp160 sequences in the two tissues in each subject. Two subjects had significantly shorter gp160 sequences in the BM (Subjects 34 and 3). When we examined the V1 to V4 region, sequences were shorter in BM than PL in four subjects (Subjects 33, 34, 1, and 3), while sequences were longer in BM in one subject (Subject 16). However, comparison of pooled PL to BM in aggregate showed that gp160 and V1 to V4 sequences from BM were not significantly different than those from PL (data not shown); the same was true when examining variable regions separately (V1, V2, V3, V4, and V5).

### Prediction of syncytium-inducing phenotype

**Figure 6:**
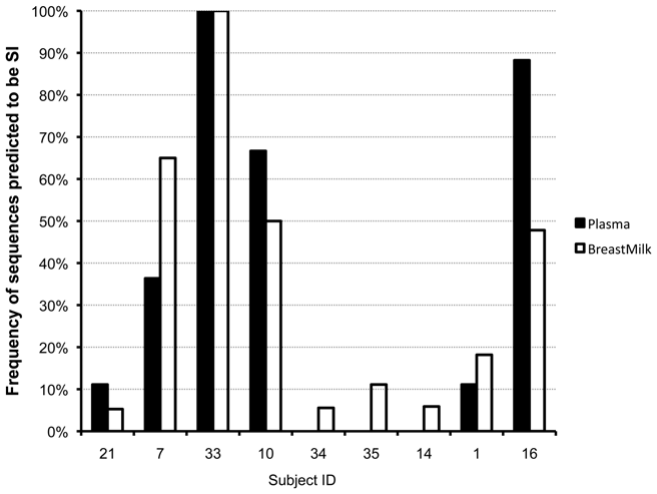
Percentage of sequences in plasma and breast milk predicted to have a syncytium-inducing (SI) phenotype. Prediction made by the Web PSSM from the V3 amino acid sequence. No SI sequences were predicted from the V3 loops in Subjects 31, 32, 17, and 3.

A subtype C position-specific scoring matrix of V3 amino acid sequences (WebPSSM) was used to predict syncytia-inducing (SI) variants. SI variants were predicted in sequences from 9 of 13 subjects (all except subjects 31, 32, 17, and 3) (Figure 6). SI variants were a minority of the viral population in four of these nine, detected in only one or two BM or PL sequences (Subjects 21, 34, 35, and 14), with SI variants predicted in BM only in three of the four. SI variants were found in over 45% of BM variants in Subjects 7, 10, and 16, while 100% of the BM and PL sequences were predicted to be SI in Subject 33. In no case were SI variants predicted in PL but not in BM.

### Phenotypic Characteristics of Plasma and Breast Milk Env

One hundred and fifty-eight clones from the PL and BM of 11 women were compared for their sensitivity to the entry inhibitors to Tak-779 and T-20. (Subjects 16 and 34 were not included). The mean IC50 of PL variants to Tak-779 was 0.0234 ug/mL (std error = 0.006) and was significantly higher than that of BM, which had a mean IC50 of 0.0165 ug/mL (std error = 0.006), (p = 0.003). However, when stratified by compartmentalization classification, sensitivity to TAK-779 was only significantly different in the compartmentalized women. No differences in susceptibility to T-20 were found overall or when the women were stratified by compartmentalization classification.

## Discussion

Defining the characteristics of HIV-1 variants in a transmitting mucosal compartment may offer important clues to understanding the nature of the genetic bottleneck observed during transmission [1], [2], [4], [5], [6], [7], [8], [38], [57]. Despite its importance in pediatric HIV infection, only a few studies have characterized HIV-1 variants in breast milk, and the results are conflicting. Two small studies reported virologic compartmentalization (6,7); however, these studies focused on a very short region of *env*, did not employ methodologies to avoid sequence resampling, and defined compartmentalization on the basis of visual inspection of trees. In contrast, a study using a heteroduplex-tracking assay (HTA), which has the ability to sample a large number of V1V2 variants [34], found no differences between PL and BM viral populations. We therefore sought to characterize HIV-1 in BM and PL in a much larger cohort and analyzed the entire *env* gene, using conditions explicitly designed to avoid sequence template resampling.

The immunologic milieu of breast milk is clearly distinct from that in blood and contains high concentrations of HIV-1 specific T cells, antibodies, cytokines, chemokines, and innate factors that modulate HIV-1 transmission risk [58], [59], [60]. Given clear immunologic compartmentalization [61] and the markedly lower amounts of HIV-1 in breast milk [14] (Figure 1), we hypothesized that virologic compartmentalization would exist between BM and PL. We amplified and cloned 356 unique, full-length gp160 *env* sequences from the BM and PL of 13 women using limiting dilution and multiple independent PCR amplifications to minimize both template resampling and PCR-product recombination. A few samples were amplified using single genome amplification approaches [62]; however, limitations in sample quantity and cost precluded widespread use of this technique. Since there is no consensus on the optimal approach for evaluating virologic compartmentalization we employed five different tests [50]. Using a majority consensus approach, only one of six subjects with contemporaneously obtained samples was classified as having compartmentalized virus (Table 2), despite an almost 100-fold difference in HIV-1 RNA levels between PL and BM.

We sought to identify factors that may have confounded our ability to detect virologic compartmentalization. Breast epithelial tight junctions are “leaky” during changes in lactation practice as well as during inflammation (mastitis). All samples were collected from women who were exclusively breastfeeding, none had a history of breast pathology, and when available, had BM sodium levels that were not markedly elevated [63]. Thus, all milk samples were obtained from women in whom breast epithelial tight junctions would be predicted to be closed. Also, the low levels of BM HIV-1 RNA support an intact breast epithelium. Recombination between parental sequences from each tissue type could also mask compartmentalization [50]. However, even when the analysis of milk sequences was restricted to regions bordered by recombination breakpoints, no evidence of compartmentalization was detected using the various tests.

Since we could only examine samples that contained relatively high levels of HIV-1, by necessity our study population was biased. In studies of temporal dynamics of breast milk HIV-1 RNA levels, at four months post-partum 57% of women in ZEBS had BM viral loads <50 copies per ml, and in those with quantifiable levels the median value was only 364 copies per ml [14]. Thus, the women included in this analysis were not “typical,” and temporal fluctuations in viral populations coupled with the relatively small numbers of clones we amplified may have confounded our ability to detect compartmentalization. Indeed, some studies indicate that compartmentalization may be more easily detected when viral loads are low, particularly in subjects who are on antiretroviral therapy compared to those who are therapy-naive [64]. Suppressing viral load could allow for variants within BM to replicate separately and appear as distinct from that in blood, while high viral loads in all tissues could cause a “swamping” of signal, in which plasma virus flooding the tissues obscures detection of within-tissue replication.

We found that sampling interval can have a striking effect on compartmentalization tests. When sequences from PL and BM samples were collected 10 or more days apart, the majority (6 of 7) were classified as compartmentalized (Table 2); if recombination was taken into account, all seven non-contemporaneously sampled subjects met criteria for compartmentalization. In addition, there was a correlation between the sampling time interval and 3 of 4 qualitative measures of compartmentalization, so that the greater the amount of time between sampling, the more frequently compartmentalization was detected (Figure 3). However, significant compartmentalization was detected even in the subjects with the smallest intervals between sampling (10 to 31 days), reflecting the high rate of HIV-1 evolution. Differences in compartmentalization were also reflected functionally in differential susceptibility to Tak-779. These analyses underscore the importance of obtaining contemporaneous samples in compartmentalization analyses. These data also highlight the importance of longitudinal studies, which could elucidate the direction and rate of viral migration between these tissues not only during lactation but also in response to inflammatory stimuli [65].

Though the difference between BM and PL viral diversity was not significant (Figure 5B), there was a trend for BM to be less diverse than PL, which could be the result of the multiple factors native to BM that may impede multi-variant outgrowth (such as antibodies, mucin, natural ligands to CCR5 that competitively inhibit HIV-1 binding, and chemokines and cytokines that create a hostile environment for HIV-1 [58], [59], [60]). Another factor that could contribute to a decrease in overall BM diversity is the presence of two or more identical or nearly identical sequences within individuals' BM. Nine subjects had from two to nine BM sequences which were identical or nearly identical (despite careful efforts to avoid resampling and contamination), which could be indicative of localized clonal bursts of virus production [15] within the BM environment immediately prior to sampling, either due to host restrictions on replication, or to transient effects of single-dose nevirapine, as has been found in subjects on suppressive ART [66], [67]. Divergence in BM was also slightly lower than in PL (Figure 5D). This difference could be indicative of a different host-immunologic response within this tissue resulting in the persistence of infected cells for longer periods – if archival sequences are able to persist in this environment they would drag the average divergence down compared to more divergent, contemporaneously circulating virus in the PL [22].

A primary focus of our study was to determine whether BM was enriched for variants that have been identified in newly infected persons. In subtype C sexual transmission, variants that establish infection have shorter variable loops, fewer potential N-linked glycosylation sites, and use CCR5 for entry [6], [8], [9], [10], [11]. We found virtually no difference between PL and BM in either PNG counts or lengths, in gp160 or by region; this overall lack of any defining feature of BM in this respect is concordant with our inability to detect compartmentalization in most subjects, and reinforces the observation that HIV-1 in breast milk appears to be very similar to that found in plasma. Newly transmitted viruses are also distinguished by almost exclusive use of CCR5. Using a subtype C phenotype-prediction method [54] we detected SI variants in the breast milk of 9 of 13 women. Thus, our data indicate that CCR5-using variants are not preferentially selected for within BM, suggesting that this tissue may not be responsible for the major bottleneck that occurs upon transmission [10].

We detected evidence for a far higher incidence of SI-using variants in our data set than initially expected. HIV-1 subtype C viruses have historically been reported at lower frequencies of CXCR4-using strains than in other group M subtypes [68], [69], [70]. A switch from R5 tropism to X4 tropism has been associated with disease progression in other subtypes [71], [72], and while this association has not been established in subtype C [73], the relatively high proportion of SI variants in our dataset may reflect a very biased population – all our subjects had advanced HIV disease and transmitted virus to their children. It may also represent an overall evolutionary change in the epidemic of subtype-C HIV-1, in which CXCR4-tropism (or CCR5/CXCR4 dual tropism) is increasing in subtype C, as has been suggested [74].

In conclusion, within the limitation on inference imposed by the number of women examined here (N = 13), the genetic bottleneck observed during HIV transmission does not appear to be mediated by selection within breast milk. Furthermore, our studies highlight HIV-1's rapid evolution and the importance of well characterized and appropriately timed sampling in both genotypic and phenotypic studies of HIV variants. Further studies defining factors that restrict HIV entry into breast milk remain important for understanding and preventing milk-borne pediatric HIV-1 transmission.
